# Phenotypic and Genotypic Bacterial Virulence and Resistance Profiles in Hidradenitis Suppurativa

**DOI:** 10.3390/ijms26083502

**Published:** 2025-04-09

**Authors:** Corina Ioana Cucu, Călin Giurcăneanu, Elena Poenaru, Liliana Gabriela Popa, Mircea Ioan Popa, Mariana Carmen Chifiriuc, Veronica Lazăr, Alina Maria Holban, Irina Gheorghe-Barbu, Andrei-Alexandru Muntean, Costin Ștefan Caracoti, Mara Mădălina Mihai

**Affiliations:** 1 Department of Oncologic Dermatology, “Elias” Emergency University Hospital, “Carol Davila” University of Medicine and Pharmacy, 020021 Bucharest, Romania; corina-ioana.cucu@drd.umfcd.ro (C.I.C.); calin.giurcaneanu@umfcd.ro (C.G.); liliana.popa@umfcd.ro (L.G.P.); mara.mihai@umfcd.ro (M.M.M.); 2 Department of Medical Informatics and Biostatistics, “Carol Davila” University of Medicine and Pharmacy, 020021 Bucharest, Romania; 3 Department of Microbiology II, “Cantacuzino” Institute, “Carol Davila” University of Medicine and Pharmacy, 020021 Bucharest, Romania; mircea.ioan.popa@umfcd.ro (M.I.P.); costin-stefan.caracoti@drd.umfcd.ro (C.Ș.C.); 4 Research Institute of the University of Bucharest-ICUB, University of Bucharest, 050663 Bucharest, Romaniaveronica.lazar@bio.unibuc.ro (V.L.); gheorghe@bio.unibuc.ro (I.G.-B.)

**Keywords:** hidradenitis suppurativa, skin microbiome, biofilm, virulence factors, *Staphylococcus aureus*

## Abstract

Hidradenitis suppurativa (HS) is a chronic inflammatory skin condition, primarily affecting young individuals, with a significant impact on their quality of life due to recurrent, painful nodules, abscesses, and oozing sinus tracts, primarily affecting intertriginous areas. The pathogenesis of HS is multifactorial, involving a complex interplay between genetic predisposition, immune dysregulation, microbial, and environmental factors. While it is known that cutaneous and gut microbiome contribute to innate immune dysregulation in HS, their precise involvement in disease pathogenesis remains unclear. Despite several studies investigating the microbiome of HS lesions, either by culture-dependent or independent methods, there is no data available on the interplay between bacterial virulence profiles, clinical manifestations, and the host immune response. This study aimed to explore the phenotypic and genotypic resistance and virulence profiles of microorganisms isolated from HS lesions (including the expression of soluble virulence factors and the ability to develop biofilms), with a special focus on *Staphylococcus aureus* (*S. aureus*), one of the most frequent infectious agents of HS. A total of 92 bacterial strains, belonging to 20 different bacterial species, were isolated from the HS lesions of 23 patients. The strains of *Staphylococcus*, *Corynebacterium*, and *Enterococcus* expressed the highest levels of soluble virulence factors, such as hemolysins, lecithinase, and lipase, which are involved in bacterial persistence, local invasivity, and tissue damage. Moreover, a significant variation among bacterial species was noted regarding the capacity to develop biofilms, with a potential impact on disease chronicization, bacterial tolerance to antibiotics, and immune defense mechanisms. The genetic characterization of methicillin-resistant staphylococci revealed the presence of adhesins, hemolysin and enterotoxin genes as well as methicillin and macrolides resistance genes. Our findings highlight the critical role of virulence determinants, including bacterial biofilms, in HS pathogenesis, emphasizing the need for targeted therapeutic strategies to disrupt biofilms and mitigate infection severity.

## 1. Introduction

Hidradenitis suppurativa (HS) is a chronic inflammatory skin condition primarily affecting young individuals with a significant impact on their quality of life [1,2,3]. Clinically, HS manifests as recurrent, painful nodules, abscesses, and oozing sinus tracts, primarily affecting intertriginous areas [1,2,3]. HS incidence is rising worldwide, partly due to high consumption of processed foods, excessive sugar intake, sedentary lifestyle, nutrient-poor diets, and hormonal imbalances [1,2,3]. A mean disease incidence of 6.0 per 100,000 inhabitants and an average prevalence of 1% have been reported in Europe [1,2,3]. In North American and European patients with HS, the female-to-male ratio is approximately 3:1, but the ratio is 1:2 in Korean patients [1,2,3].

Existing clinical classification systems, such as the Hurley classification, offer a restricted and undervalued representation of the extent of the disease. The Hurley Staging System aims to classify the severity of non-inflammatory, inactive HS, with Stage I being local nodules, Stage II showing abscesses and tunnels, and Stage III representing extensive, interconnected tunnels [1]. However, the ability to distinguish between various lesion types and accurately assess the level of disease activity is challenging.

Despite extensive research, the pathogenesis of HS remains incompletely understood. It is widely accepted that HS is the result of a complex interplay between multiple factors, genetic predisposition, immune dysregulation, hormonal influences, and microbial factors contributing to its development [4]. Bacterial colonization and biofilm formation have been recognized as key contributors to disease chronicity and recurrence [5,6,7,8]. The involvement of microbial communities in HS has gained significant attention, particularly due to the frequent isolation of *Staphylococcus* spp., *Corynebacterium* spp., and *Enterococcus* spp. from the skin lesions. Therefore, HS should be considered as part of the spectrum of disorders associated with bacterial biofilm formation [5,6]. Biofilm development represents a survival and proliferation strategy for bacteria, functioning as a primitive form of cellular differentiation [9,10]. This process creates protective environments for microorganisms, rendering them tolerant to immune system defense mechanisms and challenging to eradicate with antimicrobials [9,10,11,12,13]. Moreover, long-term treatment with antibiotics increases the risk of developing bacterial resistance and, in immune-suppressed individuals, life-threatening infections [9,10,11,12,13,14].

This study aimed to explore the phenotypic and genotypic virulence profiles of microorganisms isolated from HS lesions (including the expression of soluble virulence factors and the ability to develop biofilms). Given the high incidence of *Staphylococcus aureus* (*S. aureus*) infections in HS, along with the species potential for developing multi-drug resistance, we conducted a more in-depth study of the phenotypic and genotypic virulence profiles of *S. aureus* strains, as well as the characterization of resistance genes in methicillin-resistant staphylococci.

Understanding the complex interplay between microbial biofilms, virulence factors, and host immune responses in HS is crucial for developing targeted therapeutic interventions. By elucidating the microbial dynamics within HS lesions, this study seeks to contribute to the growing body of knowledge on HS pathogenesis and provide insights into potential strategies for improving disease management and patient outcomes.

## 2. Results

### 2.1. Study Group Characteristics

**Figure 1:**
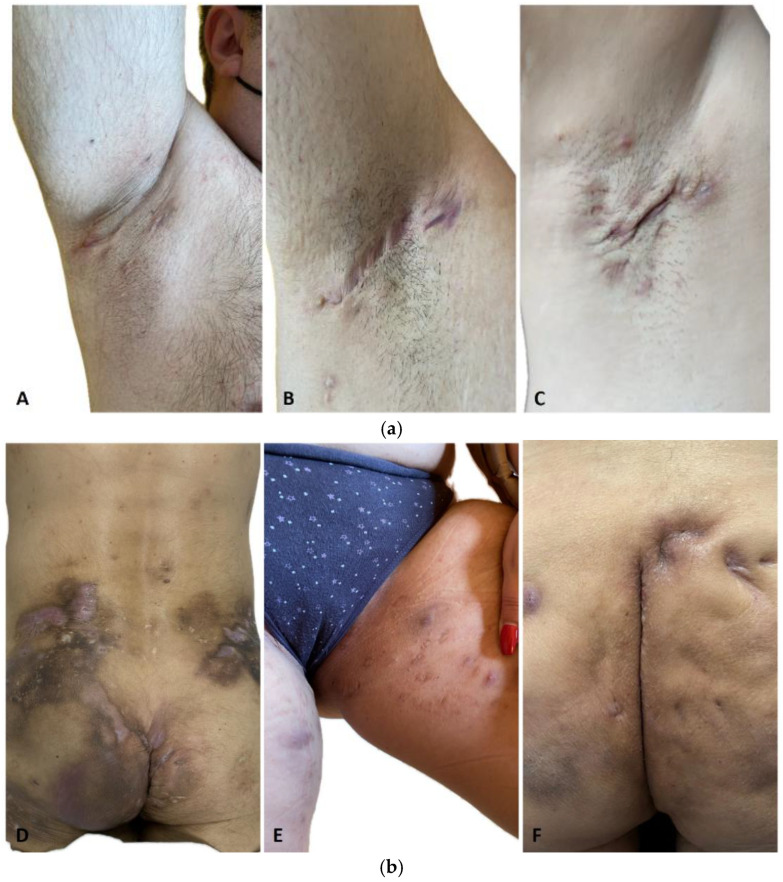
Axillary HS in different male patients included in this study: (**a**) (**A**). HS, Hurley stage I—inflammatory nodules without skin tunnels or scarring; (**B**). HS, Hurley stage II—recurrent abscesses with skin tunnels and scarring, single or multiple widely separated lesions; (**C**). HS Hurley stage III—diffuse or almost diffuse involvement, or multiple interconnected skin tunnels and abscesses across the entire affected area (**b**) (**D**). Severe gluteal HS with multiple fistulous tracts in a male patient (**E**). Inguinal HS in a female patient (**F**). Gluteal HS with several inflammatory nodules and scars in a male patient.

A total number of 23 patients diagnosed with chronic HS were enrolled in this study, all of them originating from urban areas. A total of 15 patients were males (65.3%) and 8 were females (34.7%); their mean age was 36.1 ± 11.7 years. A total of 21 patients were active smokers. A total of 23 patients were assessed, and the following numbers were observed: 4 patients (19%) had normal weight, 3 patients (14%) were overweight, 6 patients (26%) were diagnosed with class 1 obesity, 4 patients (17%) with class 2 obesity, and 6 patients (24%) with class 3 obesity. 13 patients (56%) were classified as Hurley stage III, 7 patients (30%) as Hurley stage II, and 3 patients (13%) as Hurley stage I (Figure 1).

### 2.2. Microbiological Characterization

#### 2.2.1. Bacterial Strains Identification

A total number of 92 bacterial strains of 20 different bacterial species were isolated from the lesions of 23 patients diagnosed with chronic HS. Most of these strains were isolated from the axillary region, accounting for 51 strains (55%), followed by the gluteal region with 17 strains (19%), the inguinal region with 16 strains (17%), the abdominal area, and the nuchal region, where 5 strains (6%) and 3 strains (3%), respectively, were identified (Table 1).

The most prevalent species identified were *Staphylococcus epidermidis* (*S. epidermidis*) (28%) and *S. aureus* (15%), whereas *Acinetobacter nosocomialis* (1%, *A. nosocomialis*), *Brevibacterium celere* (1%, *B. celere*), and *Corynebacterium coyleae* (1%, *C. coyleae*) were the least common. Details regarding the isolated bacterial strains, distribution by Hurley stage, and cutaneous localization of the skin lesions for the isolated stains are depicted in Table 1.

The correlation between the sampling site and the bacterial species identified was assessed using the Chi-Square tests. A statistically significant association was found (likelihood ratio 0.041 < 0.05). A statistically significant association was also demonstrated between the Hurley stage and the bacterial species isolated (likelihood ratio 56.364 with a significance level of 0.028 < 0.05).

#### 2.2.2. In Vitro Assessment of Biofilm Development 

**Figure 2:**
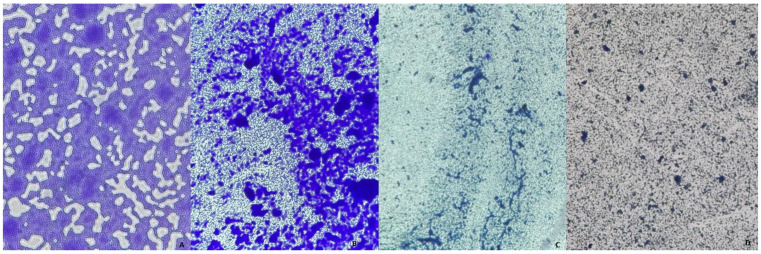
Biofilms of (**A**) *Proteus mirabilis*; (**B**) *Alcaligenes faecalis*; (**C**) *Staphylococcus lugdunensis*; (**D**) *Micrococcus luteus* at 24 h under the optical microscope at 20× magnification.

All bacterial strains isolated from HS lesions demonstrated the ability to form biofilms on inert substrata, with the extent of biofilm biomass varying by species (Figure 2).

**Figure 3:**
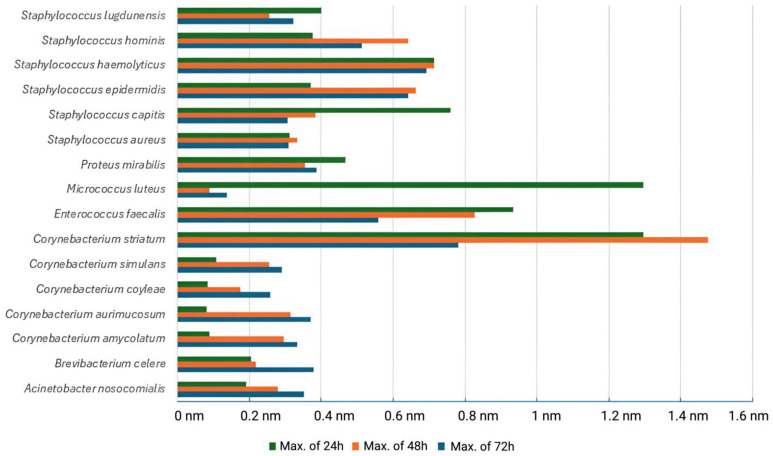
Biofilm forming capacity of isolated bacterial species at 24, 48, and 72 h.

Absorbance values obtained via crystal violet staining were directly proportional to the quantity of biofilm formed. At 24 h, *Corynebacterium* (*C.*) *striatum* exhibited the highest biofilm production (mean absorbance: 1.02 nm), followed by *Enterococcus* (*E.*) *faecalis* and *Micrococcus* (*M.*) *luteus*. In contrast, *Corynebacterium* (*C.*) *aurimucosum* showed the lowest biofilm biomass (0.06 nm). At 48 h, *C. striatum* remained the most prolific biofilm-forming strain (1.43 nm), while *M. luteus* continued to show minimal biomass accumulation (0.09 nm). Several other strains, including *Corynebacterium* (*C.*) *simulans* and *Enterococcus faecalis*, also demonstrated strong biofilm growth. By 72 h, a general decline in absorbance values was observed for most species. *C. striatum* still led in biofilm formation (0.66 nm), whereas *Acinetobacter* (*A.*) *nosocomialis* and *Corynebacterium* (*C.*) *amycolatum* showed only modest activity. This reduction may be attributed to biofilm maturation and partial detachment from the microplate wells during fixation and staining. However, for the remaining *Corynebacterium* species, *Brevibacterium celere*, and *A. nosocomialis*, biofilm formation continued to increase at 72 h. This ascending trend may be attributed to the slower biofilm development rate of these strains compared to the others tested. Overall, the most substantial biofilm development occurred within the first 24–48 h, highlighting the early biofilm-forming potential of certain strains—particularly *C. striatum*—isolated from HS lesions (Figure 3).

**Figure 4:**
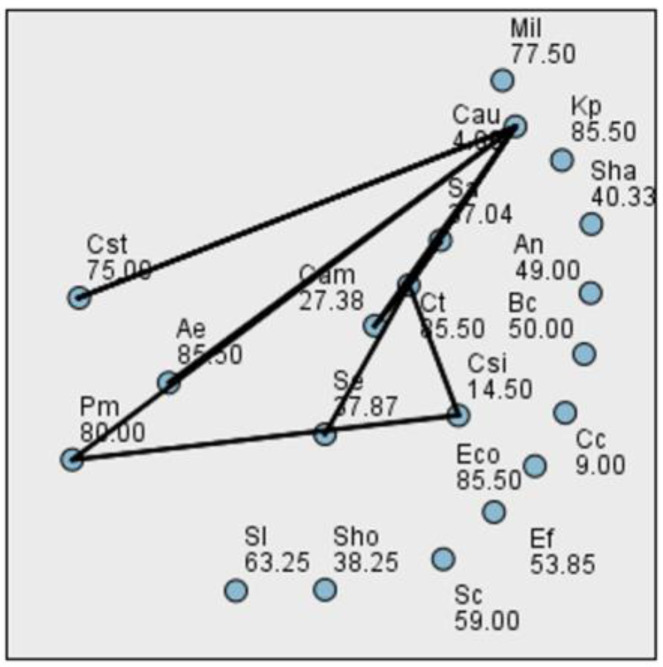
Pairwise comparison between bacterial species biofilm production at 24 h.

**Figure 5:**
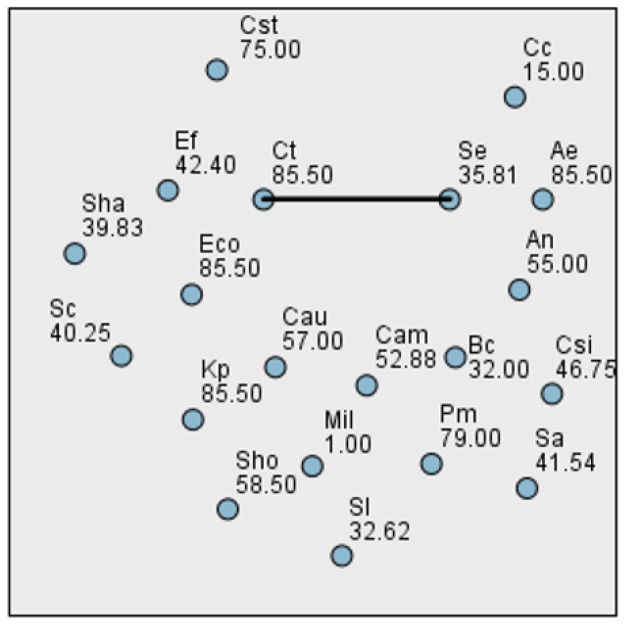
Pairwise comparison between bacterial species biofilm production at 48 h.

**Figure 6:**
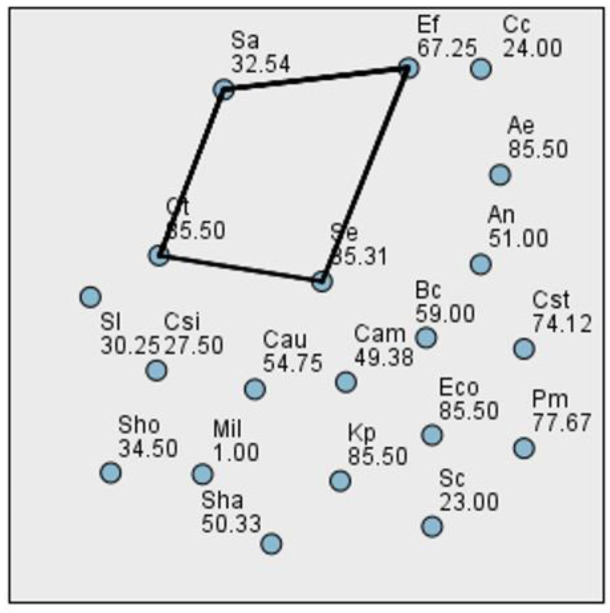
Pairwise comparison between bacterial species biofilm production at 72 h.

To assess the differences in biofilm production across bacterial species at various time points, we first tested the distribution of the data using the Kolmogorov–Smirnov and Shapiro–Wilk normality tests. Although several *p*-values were greater than 0.05, the data at all three time points deviated from a normal distribution. Consequently, we employed the Kruskal–Wallis test, a non-parametric alternative to one-way ANOVA, suitable when assumptions of normality and homogeneity of variance are not met. At 24 h, the Kruskal–Wallis test yielded a statistic of H = 45.661 with 19 degrees of freedom and a *p*-value of 0.0005535, indicating no statistically significant differences in biofilm formation among the tested bacterial species (Figure 4). At 48 h, the data remained non-normally distributed. The Kruskal–Wallis test revealed H = 36.553, df = 19, and a *p*-value of 0.009022, indicating statistically significant differences in biofilm biomass between bacterial species (Figure 5). At 72 h, the Kruskal–Wallis test result was H = 46.976, df = 19, and *p* = 0.000360, once again indicating no significant differences between bacterial strains in terms of biofilm development at this time point (Figure 6). This result may reflect the general decrease in biomass at 72 h, likely due to biofilm detachment during processing.

Biofilm formation significantly contributes to bacterial persistence due to increased tolerance to antimicrobials as well as to host immune response mechanisms. Identifying the ability to develop biofilms of specific strains allows for targeted therapeutic approaches, potentially improving patient outcomes by employing treatments that disrupt biofilms and enhance bacterial eradication. Understanding the biofilm-forming capacity of different bacterial strains in HS is crucial for developing effective treatment strategies.

#### 2.2.3. Phenotypic Assessment of the Production of Soluble Virulence Factors

**Figure 7:**
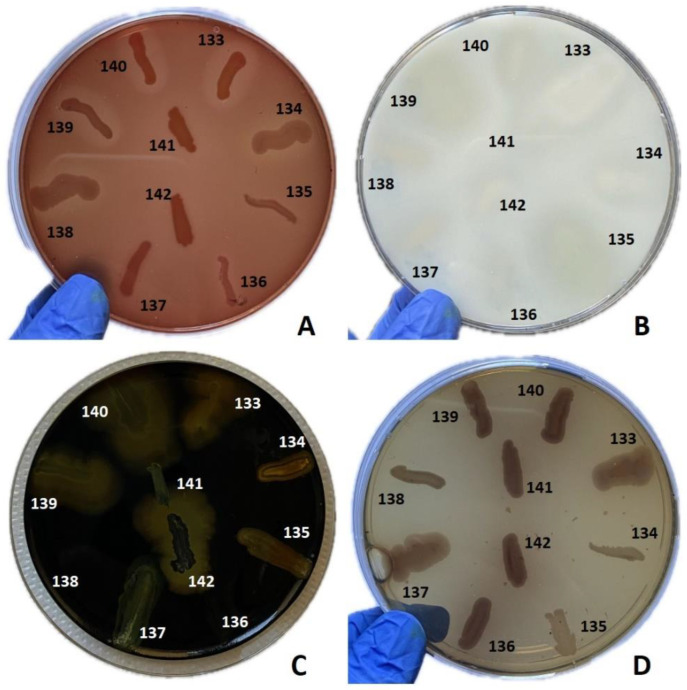
Assessment of the production of different virulence factors in 10 bacterial strains analyzed. (**A**) Virulence factor: hemolysins, medium: agar supplemented with 5% sheep blood; (**B**) virulence factor: caseinase, medium: agar with addition of 15% casein; (**C**) virulence factor: amylase, medium: agar with 1% starch added, flooded with Lugol solution; (**D**) virulence factor: esculinase, medium: agar supplemented with 1% esculin and iron citrate. Bacterial strains tested: no. 133 *Staphylococcus epidermidis* (*S. epidermidis*), no. 134 *S. epidermidis*, no. 135 *S. epidermidis*, no. 136 *S. epidermidis*, no. 137 *S. epidermidis*, no. 138 *Staphylococcus* aureus, no. 139 *S. epidermidis*, no. 140 *Enterococcus faecalis*, no. 141 *Corynebacterium amycolatum*, no. 142 *Corynebacterium aurimucosum*.

**Figure 8:**
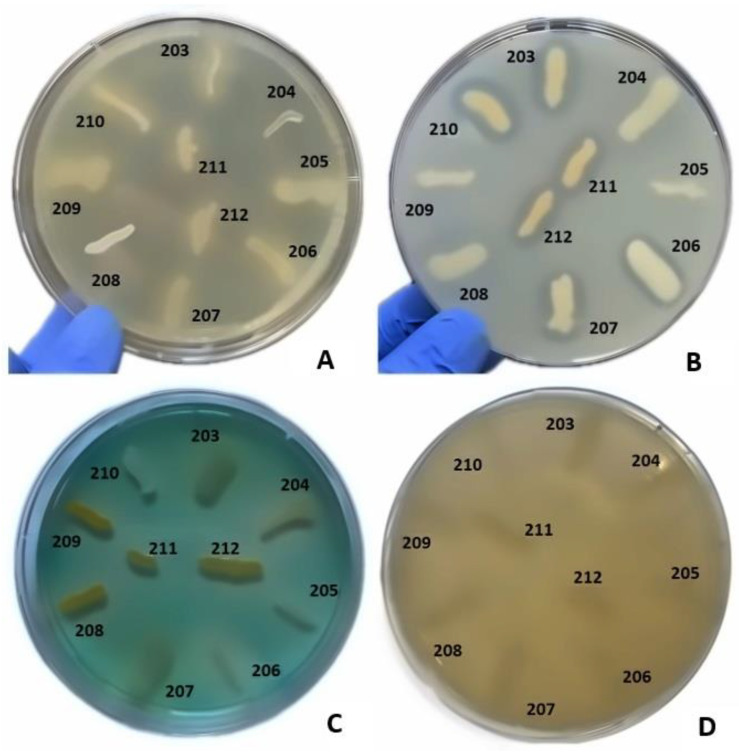
Assessment of the production of different virulence factors in 10 bacterial strains analyzed. (**A**) Virulence factor: lipase, medium: agar supplemented with 1% Tween 80; (**B**) virulence factor: lecithinase, medium: agar with the addition of 2.5% egg yolk; (**C**) virulence factor: DNase, medium: DNase; (**D**) virulence factor: gelatinase; medium: agar with the addition of 3% gelatin. Bacterial strains tested: no. 203 *Staphylococcus aureus* (*S. aureus*), no. 204 *S. aureus*, no. 205 S. aureus, no. 206 *S. haemolyticus*, no. 207 *Corynebacterium striatum* (*C. striatum*), no. 208 *C. striatum*, no. 209 *Staphylococcus epidermidis*, no. 210 *Staphylococcus epidermidis*, no. 211 *C. striatum*, no. 212 *C. striatum*.

The production of soluble virulence factors across bacterial genera was analyzed and compared by the cultivation of isolated strains on specialized media supplemented with enzyme substrates specific to each soluble virulence factor. These factors included pore-forming toxins (lecithinase, lipase, hemolysins) and exoenzymes (caseinase, gelatinase, amylase, DN-ase and esculinase). The distribution of virulence factors in the isolated bacterial strains is presented in Table 2, Figure 7 and Figure 8.

In *Staphylococcus* sp. strains, the most frequently expressed virulence factors were hemolysins (53 strains, 94.64%), followed by lecithinase (detected in 35 strains at 72 h, 62.5%), and lipase (23 strains at 72 h, 41%). Lower expression levels were noted for caseinase (6 strains, 10.71%), and amylase (7 strains, 12.5%). Gelatinase and DN-ase exhibited no expression among the analyzed strains. These findings indicate that *Staphylococcus* strains predominantly produce pore-forming toxins (hemolysins, lecithinase, and lipase), which play a crucial role in bacterial proliferation and dissemination. Hemolysins contribute to the mobilization of iron, which is essential for the activation of microbial genes and the subsequent expression of additional virulence factors.

In *Corynebacterium* strains, the most frequently expressed virulence factors were hemolysins (12 strains, 75%), followed by caseinase (6 strains, 37.5%) and lecithinase (detected in 5 strains at 72 h, 31.25%). Lipase expression was detected in three strains at 72 h, (18.75%). Lower expression levels were noted for esculinase, while amylase, gelatinase, and DN-ase exhibited no expression among the analyzed strains. Hemolysins were the most common virulence factor expressed in both *Staphylococcus* and *Corynebacterium* strains, but the *Staphylococcus* strains showed significantly more intense lecithinase (2:1) and lipase (2.18:1) expression.

*Enterococcus* strains mostly expressed esculinase (nine strains, 90%), caseinase (six strains, 60%), and lipase (five strains, 50%), but none of the strains showed complete hemolysis, lecithinase, DN-ase, or gelatinase expression.

#### 2.2.4. Genotypic Assessment of Virulence and Resistance Genes of *Staphylococcus aureus* Strains by PCR

Phenotypic changes are correlated with a certain genotypic profile. Considering this, genotypic screening of the analyzed strains aimed to identify the presence of several genes encoding extracellular virulence factors or involved in bacterial invasiveness and adhesion. The genotypic virulence profile was investigated in 35 *S. aureus* strains (Table 3), while the genotypic resistance profile was analyzed in 12 strains (Table 4).

**Figure 9:**
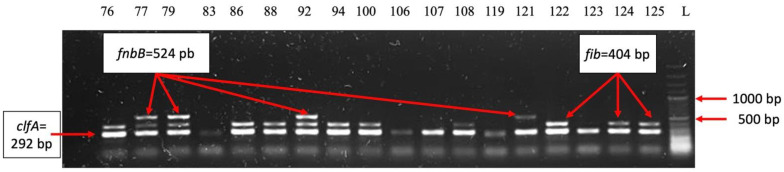
Electrophoresis of DNA amplicons PCR for the genes *fnbB* (524 bp), *fib* (404 bp), *clfA* (292), *clfB* (205 bp). Lines 2–19-strain 76; 77; 79; 83; 86; 88; 92; 94; 100; 106; 107; 108; 119; 121; 122; 123; 124; 125. Line 20-Molecular size marker (Thermo Scientific, Waltham, MA, USA, 1500 bp). Strains positive for the *fib* gene: 76; 77; 79; 86; 88; 92; 94; 100; 108; 122; 124; 125. Strains positive for the *clfA* gene: 76; 77; 79; 83; 86; 88; 92; 94; 100; 106; 107; 108; 119; 121; 122; 123; 124; 125. Strains positive for the *fnbB* gene: 77; 79; 92; 121.

**Figure 10:**
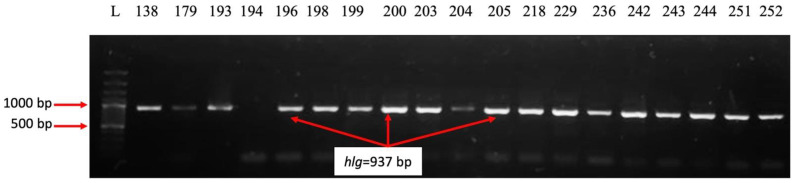
Electrophoresis of DNA amplicons post PCR for the *luk-PV* (443 bp) and *hlg* (937 bp) genes. Line 1-Molecular size marker (Thermo Scientific, Waltham, MA, USA, 1500 bp); lines 2–20 strains 138; 179; 193; 194; 196; 198; 199; 200; 203; 204; 205; 218; 229; 236; 242; 243; 244; 251; 252. Positive strains for the *hlg* gene: 138; 179; 193; 196; 198; 199; 200; 203; 204; 205; 218; 229; 236; 242; 243; 244; 251; 252.

**Figure 11:**
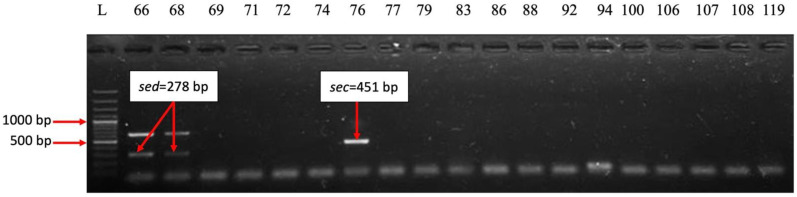
Electrophoresis of DNA amplicons post PCR for genes *sea* (102 bp), *seb* (164 bp), *sec* (451 bp), *sed* (278 bp), *see* (209 bp). Line 1-Molecular size marker (Thermo Scientific, Waltham, MA, USA 1500 bp); lines 2–20 strains: 68; 69; 71; 72; 74; 76; 77; 79; 83; 86; 88; 92; 94; 100; 106; 107; 108; 119. Strains positive for the *sec* gene: 76. Strains positive for the *sed* gene: 66; 68.

The analysis of the distribution of virulence genes demonstrated that 34 of the 35 strains analyzed (97.14%) expressed genes encoding adhesins associated with the bacterial surface *clfA* [13,15]. This gene helps *S. aureus* bind fibrinogen and complement regulator I protein and enables the clumping of blood plasma [16]. Fibronectin-binding proteins are *S. aureus* cell surface-bound proteins that bind to fibronectin and fibrinogen [17], making it adhere to host cells and facilitating colonization [13,18]. The fibrinogen-binding protein gene was present in 71.42% of strains. From the fibronectin-binding proteins, the *fnbA* gene was present in 88.57% of the strains, while the *fnbB* gene was less frequently detected (14.28%) (Figure 9). The hemolysin-*hlg* gene was present in 71.42% of strains (Figure 10), the elastin-binding protein was noticed in 45.71% of strains, the bone sialoprotein-binding protein in 5.71% of strains, and the toxic shock syndrome toxin gene in 2.85% of strains. From the classical staphylococcal enterotoxin genes, only the *sec* gene was positive in 8.57% and *sed* in 2.85%; *sea*, *seb*, *see* remained negative (Figure 11). None of the *S. aureus* strains analyzed expressed collagen-binding proteins gene and exotoxin Panton–Valentine leucocidin gene.

**Figure 12:**
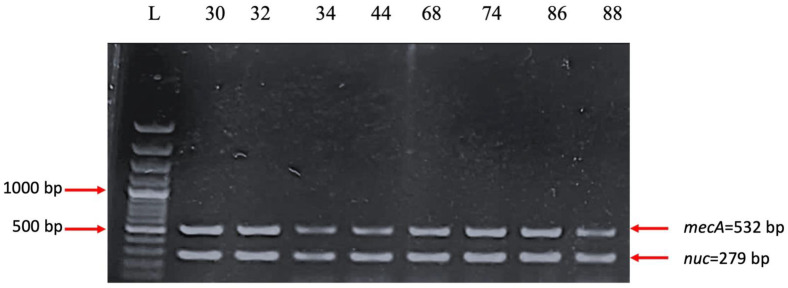
Electrophoresis of DNA amplicons post-PCR for the mecA (532 bp) and nuc (279 bp) genes—genotypic confirmation of the MRSA phenotype. Line 1-Molecular size marker (Thermo Scientific, Waltham, MA, USA, 1500 bp); lines 2–9 strains: 30; 32; 34; 44; 68; 74; 86; 88 and negative control. Strains positive for the *mecA* and *nuc* genes: 30; 32; 34; 44; 68; 74; 86; 88.

**Figure 13:**
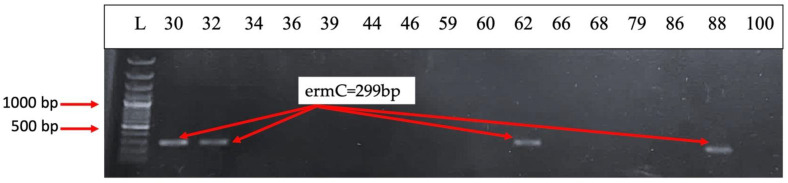
Electrophoresis of DNA amplicons post PCR for the ermA (190 bp) and ermC (299 bp) genes—macrolide resistance. Line 1-Molecular size marker (Thermo Scientific, Waltham, MA, USA 1500 bp); lines 2–17 strains: 30; 32; 34; 36; 39; 44; 46; 59; 60; 62; 66; 68; 79; 86; 88; 100. Strains positive for the ermC gene: 30; 32; 62; 88.

**Figure 14:**
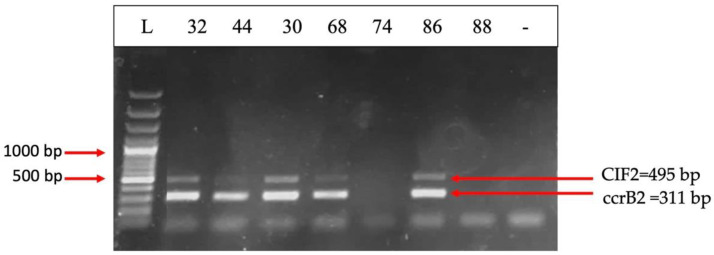
Electrophoresis of DNA amplicons post PCR for the CIF2 (495 bp) and ccrB2 (311 bp) genes from the staphylococcal chromosomal cassette SCCmec. Line 1-Molecular size marker (Thermo Scientific, Waltham, MA, USA, 1500 bp); Lines 2–8 strains: 32; 44; 30; 68; 74; 86; 88. Strains positive for the CIF2 and ccrB2 genes: 39; 32; 44; 30; 68; 86.

*The mecA* (encoding penicillin-binding protein 2a) and *nuc* (encoding thermonuclease) genes are both present in 50% of the strains (Figure 12). PBP2A is a transpeptidase that helps form the bacterial cell wall and allows it to be resistant to antibiotics such as methicillin [19], penicillin, and other penicillin-like antibiotics. Therefore, the most common *S. aureus* strain known to carry *mecA* is methicillin-resistant *S. aureus* (MRSA) [20]. A total of 25% of the strains harbored *ermC* (erythromycin ribosomal methylase which encodes resistance genes for erythromycin), while *ermA* was absent [21] (Figure 13). The *ccr* gene complex encodes the recombinases responsible for its mobility, as well as the excision and integration of *SCCmec* within the bacterial chromosome [22]. *ccrB2* and *CIF2* are present in 41.66% of the strains, but *ccrC* is present in none of them (Figure 14).

## 3. Discussion

The findings of this study highlight the significant role of bacterial biofilms and virulence factors in the pathogenesis and chronicity of HS. The results confirm that biofilm formation is a key survival strategy for bacterial strains isolated from HS lesions, contributing to persistent infections, resistance to antimicrobial treatment, and prolonged inflammation [23]. Biofilm-associated bacteria exhibit distinct phenotypic characteristics compared to their planktonic counterparts, including altered virulence factor expression and heightened resistance to conventional antimicrobial treatments [6,24]. Furthermore, the phenotypic and genotypic analysis of virulence factors in *Staphylococcus* spp., *Corynebacterium* spp., and *Enterococcus* spp. reveals distinct patterns of pathogenicity that may exacerbate disease severity, perpetuating immune activation, impairing wound healing, and hindering effective treatment [25]. Also, commensal bacteria may elicit inflammatory responses in genetically susceptible individuals [26]. Their ability to form robust biofilms suggests their active role in the chronic nature of HS lesions. Biofilm-associated bacteria exhibit altered gene expression and metabolic states, rendering them highly resistant to antibiotics and immune responses [27]. The crystal violet microtiter plate assay demonstrated a wide variability in biofilm formation among different species, with *C. striatum* exhibiting the highest biofilm biomass production. These findings align with previous studies suggesting that *Corynebacterium* spp. are prominent contributors to chronic skin infections due to their strong biofilm-forming capabilities [9,28,29]. Bacteria within biofilms evade host immune responses by resisting phagocytosis and producing virulence factors that modulate the local inflammatory environment. In addition to biofilm formation, the production of virulence factors was significantly associated with bacterial persistence and pathogenicity [30]. The predominance of hemolysins, lecithinase, and lipase among *Staphylococcus* strains indicates their potential to cause tissue damage, evade immune responses, and facilitate bacterial dissemination [31]. Hemolysins, in particular, play a crucial role in iron acquisition, which is essential for bacterial survival and virulence gene activation.

Moreover, genotypic analyses confirmed the presence of virulence genes encoding adhesins and extracellular toxins, further reinforcing the pathogenic potential of *S. aureus* in HS lesions. Notably, resistance gene analysis in MRSA, strains revealed the presence of *mecA* and *ermC*, conferring resistance to beta-lactam and macrolide antibiotics, respectively. The presence of *mecA* in 50% of analyzed strains confirms the high prevalence of MRSA in chronic HS lesions, necessitating alternative treatment approaches, such as biofilm-targeted therapies or combination antibiotic regimens.

The clinical implications of these findings are substantial. Given the confirmed role of biofilms in HS chronicity, therapeutic strategies should focus on disrupting biofilm integrity through mechanical debridement, enzymatic agents, or combination antibiotic therapies that target both planktonic and biofilm-associated bacteria. Additionally, the identification of key virulence factors provides insight into potential therapeutic targets, such as inhibitors of hemolysins or adhesion molecules, to reduce bacterial pathogenicity and improve treatment outcomes.

Despite the valuable insights provided by this study, some limitations should be acknowledged, which could be addressed in future research. First, the relatively small sample size of patients may limit the generalizability of the findings to a broader HS population. Moreover, a larger and more diverse patient cohort, ideally incorporating samples from multiple clinical centers, would enhance the generalizability of our findings. Another limitation is the reliance on in vitro biofilm formation assays, which may not fully replicate the in vivo conditions within HS lesions. Future studies with larger cohorts, advanced metagenomic analysis, and in vivo biofilm models are needed to further elucidate the complex microbial interactions in HS and develop more effective therapeutic strategies.

## 4. Materials and Methods

In this observational and prospective study, we included 23 patients diagnosed with chronic HS at the “Elias” Emergency University Hospital, located in Bucharest, Romania, over the preceding four years. Prior to study initiation, informed consent was duly obtained from each patient. This study’s methodology was meticulously designed to align with the ethical mandates delineated in the 1975 Declaration of Helsinki, as well as the Good Clinical Practice (GCP) standards, receiving the requisite approval from the Ethics Commission of our medical institution. Analytical procedures were performed at the Research Institute of the University of Bucharest, Romania and the Cantacuzino National Medico-Military Institute for Research and Development, Bucharest, Romania. The identification of bacterial strains was authenticated through classical microbiological phenotypic methods. Additionally, we conducted an evaluation of the bacterial strains’ phenotypic and genotypic virulence attributes.

### 4.1. Clinical Data

The diagnosis was ascertained through physical evaluation based on the distinctive clinical presentation, which included inflammatory nodules, comedones, and fistulous lesions situated in intertriginous regions, coupled with a chronic trajectory exceeding three months in duration [1,32,33]. The inclusion and exclusion criteria of the study are referred in Table 5 and Table 6. Clinical data were amassed via anamnesis, encompassing the age of onset of HS, lesion localization, HS severity staging, comorbidities, familial medical history, smoking status, body mass index (BMI), prior treatments, and quality of life assessments [1,3,34,35]. Additionally, a comprehensive physical examination was conducted, and clinical photographs were systematically captured. 

### 4.2. Microbiological Characterization

#### 4.2.1. Bacterial Strains Identification

Specimen collection was conducted using sterile swabs from superficial purulent lesions (superficial situs) without prior skin asepsis. These samples were promptly placed in a rapid transport buffer for immediate plating on various culture media (Thioglycollate Broth with Resazurin THIO-T, bioMérieux, Craponne, France). For non-suppurative, closed lesions, access was gained via punch biopsies (profound situs), each preceded by skin asepsis using a 10% povidone–iodine solution. The selection of lesions for sampling was based on the degree of discomfort they caused the patient, assessed by inflammation, pain, recurrence, and suppuration levels. The most active lesion was chosen for bacterial sampling. Microbial cultures were subjected to both aerobic and anaerobic culture techniques. Gram staining was performed on all specimens. To isolate pure colonies, each swab was inoculated onto 5% sheep blood agar, Chocolate agar, MacConkey agar (lacking crystal violet), and Sabouraud agar with chloramphenicol (Oxoid, Cheshire, WA, USA). All plates, except for those on Sabouraud agar, were incubated aerobically at 37 °C for 18–24 h. The Sabouraud plates were incubated at both 30 °C and 37 °C for 24–72 h. Biochemical identification of the strains was carried out using the automated Vitek 2 system (bioMérieux) and Phoenix BD (Bekton–Dickinson, Franklin Lakes, NJ, USA) [13,36]. After identification, strains were preserved at 3 °C within the Microbial Culture Collection of the Microbiology Laboratory at the Faculty of Biology, Bucharest. For subsequent experiments, strains were streaked onto nutrient agar and incubated overnight at 37 °C.

#### 4.2.2. Phenotypic Assessment of Biofilm Formation In Vitro

The biofilm production capability of the isolated strains was assessed using the crystal violet microtiter plate method, which involves the spectrophotometric measurement of the biomass adhered to the inert substrate within 96-well plates. The bacterial cultures, following overnight incubation, were diluted in Tryptic Soy Broth to achieve a turbidity equivalent to 0.5 McFarland standard (approximately 1.5 × 10^8^ CFU/mL). Subsequently, 20 μL of this bacterial suspension was inoculated into 96-well plates containing 180 μL of the broth medium, with the process being replicated twice. To facilitate biofilm development, the inoculated plates underwent incubation for 24, 48, and 72 h at a temperature of 37 °C. Post-incubation, the biofilms were subjected to a gentle PBS wash to eliminate planktonic cells. The biofilm mass that remained adherent was fixed using cold methanol for 5 min, air-dried at ambient temperature, stained with a 0.1% crystal violet solution for 15 min, solubilized in 33% acetic acid, and the absorbance was measured spectrophotometrically at 492 nm. The absorbance values obtained are directly proportional to the quantity of biofilm biomass [13,37,38,39].

#### 4.2.3. Phenotypic Assessment of Production of Soluble Virulence Factors

The phenotypic expression of bacterial virulence was evaluated through enzymatic assays targeting eight soluble factors, employing specific media for each test: 5% sheep blood agar for alpha and beta hemolysins, 2.5% yolk agar for lecithinase, 1% Tween 80 agar for lipase, 15% casein agar for caseinase, 1% gelatin agar for gelatinase, 10% starch agar for amylase, DNA agar for DN-ase, and 1% esculin iron salts for esculinase [39,40,41,42]. These media were augmented with a nutritive agar base supplemented with various substrates and biochemical indicators to facilitate the detection of specific bacterial enzymes. A bacterial inoculum was prepared from a 24 h culture to a turbidity of 0.5 McFarland standard (1.5 × 10^8^ CFU/mL) and was spotted using a 10 μL sterile loop onto Petri dishes containing the respective media. The strains were incubated at 37 °C for 24 h and subsequently at 25 °C for an additional 48 h, enabling production and subsequent observation of specific enzymatic virulence factors. The evaluation of these factors was conducted after 24 and 72 h of incubation. The outcomes were quantified by monitoring the alterations in the culture medium’s appearance post-enzymatic reaction: the hemolytic zone around the inoculation site indicated alpha (incomplete hemolysis) and beta hemolysins (complete hemolysis) activity; the opaque/precipitation zone denoted the production of lecithinase, lipase, caseinase, and gelatinase (hydrolysis); a color shift from emerald green to pink around the culture spot signified DN-ase activity; black precipitation of iron ester marked esculinase activity; amylase (starch hydrolysis) activity was confirmed post-iodine solution application by a yellow ring around the inoculated strain with the rest of the medium remaining blue. The production of virulence factors was scored on a scale from 0 to 4, with 0 being the minimum and 4 the maximum, based on the diameter of the altered area in the culture medium surrounding the inoculation site.

#### 4.2.4. Genotypic Assessment of Virulence and Resistance Genes of *Staphylococcus aureus* Strains by RCR

The genotypic virulence profiles of 35 *S. aureus* strains were determined through PCR and the resistance profile was analysed for 12 selected strains. The molecular reactions were conducted using a Corbet Thermal Cycler, and the amplification products from each PCR assay (simplex or multiplex) were visualized via electrophoresis on 1% agarose gel. These gels were stained with SYBR Safe DNS (Thermo Scientific, Waltham, MA, USA) and the products were identified by their characteristic sizes in comparison to specific molecular weight markers (M-Bench Top 100 bp DNA Ladder, Promega, Madison, WI, USA) [13,38]. Genomic DNA from the bacteria was extracted using the alkaline lysis method. Colonies of MRSA (1–5) were resuspended in a solution of 20 μL NaOH (0.05 M) and sodium dodecyl sulfate (0.25%). The amplification step was carried out at 95 °C for 15 min, followed by the addition of 180 μL TE1x buffer to each tube and centrifugation at 13,000 rpm for 3 min. The purity and concentration of the resultant DNA were verified by electrophoresis on 1.5 and 2% agarose gels at 90 V for 45 min, stained with SYBR Safe DNS. The extracted genomic DNA was stored at −4 °C and utilized as a template for all subsequent PCR experiments. The genotypic virulence profile for *S. aureus* was characterized by the presence of 17 genes encoding virulence factors, which were expressed in the selected strains. The protocol delineated by Cotar et al. [43] was adhered to, maintaining the integrity of the primer sequences and the reaction parameters. The detection of several virulence genes was accomplished via Polymerase Chain Reaction (PCR) through uniplex/simplex assays (*fnbA* gene for fibronectin adhesin A; *coag* gene for coagulase enzyme) and multiplex assays (*clfA* and *clfB* genes for bacterial surface adhesins; *fnbB* gene for fibronectin-binding protein B; *fib* gene for fibrinogen-binding protein; *bbp* gene for bone sialoprotein-binding protein; *ebpS* gene for elastin-binding protein); *luk-PV* gene for leucocidin genes; *hlg* gene for hemolysin genes; *tst* gene for toxic shock syndrome toxin gene; *sea*, *seb*, *sec*, *sed*, and *see* genes for classical staphylococcal enterotoxin genes. A total of 20 genes were tested, including the m*ecA* gene (encoding penicillin-binding protein 2a); *nuc* (encoding thermonuclease); *ermA* and *ermC* (encoding resistance genes for erythromycin); *Staphylococcal* cassette chromosome (SCC); SCC*mec* group; *ccr* (which encodes the recombinases responsible for its mobility); *ccrC* and *ccrB2* (cassette chromosome recombinase); *Kdp* (high-affinity K^+^ transporter functioning as an emergency scavenger when extracellular K^+^ concentrations are low). The reaction mixture employed was the GoTaq^®^ Green Master Mix (Jena Bioscience, Jena, Germany) [43].

### 4.3. Statistical Analysis

A comprehensive statistical analysis was conducted to assess the association between the Hurley stage of hidradenitis suppurativa and various bacterial species isolated. Statistical computations were performed utilizing IBM^®^ SPSS^®^ software edition 30.0.0. The analysis included both parametric and non-parametric tests to ensure robustness and accuracy. Initially, the Shapiro–Wilk test was employed to assess the normality of the data. Consequently, the Kruskal–Wallis test, a non-parametric alternative to ANOVA, was used to compare the distributions of the bacterial species across the data. Additionally, Levene’s test was performed to evaluate the homogeneity of variances. The correlation between variables was tested by estimating the Pearson Chi-Square linear correlation coefficients according to the established criteria. Descriptive statistics, including mean, standard deviation, and confidence intervals, were also calculated to provide a detailed summary of the data for each bacterial species.

## 5. Conclusions

In conclusion, this study underscores the complex interplay between microbial biofilms, virulence factors, and antibiotic resistance in HS pathogenesis. The results revealed that all isolated strains from HS lesions developed biofilm on the inert substratum, with variations depending on the bacterial species. The high prevalence of biofilm-forming bacteria, coupled with the expression of virulence determinants, emphasizes the need for innovative therapeutic strategies aimed at biofilm disruption and targeted antimicrobial therapy. Future research should focus on the development of anti-biofilm agents and novel treatment modalities that address both bacterial persistence and host immune responses to improve patient outcomes in HS management.

## Figures and Tables

**Table 1:** Distribution of the bacterial strains isolated from HS lesions, by Hurley stage and location.

No.	Bacterial Species	No. Strains	Hurley Stage			Location				
			I	II	III	Axillary	Abdominal	Gluteal	Inguinal	Nuchal
1	*Staphylococcus epidermidis* (28%)	26	4	6	16	17	1	2	3	3
2	*Staphylococcus aureus* (15%)	14	-	4	10	6	3	5	-	-
3	*Enterococcus faecalis* (11%)	10	-	7	3	1	-	5	4	-
4	*Staphylococcus haemolyticus* (7%)	6	-	2	4	3	-	1	2	-
5	*Staphylococcus hominis* (4%)	4	-	2	2	1	1	1	1	-
6	*Staphylococcus lugdunensis* (4%)	4	-	4	-	-	-	-	4	-
7	*Corynebacterium striatum* (4%)	4	-	-	4	4	-	-	-	-
8	*Corynebacterium amycolatum* (4%)	4	-	-	4	4	-	-	-	-
9	*Proteus mirabilis* (3%)	3	-	-	3	1	-	2	-	-
10	*Corynebacterium tuberculostearicum* (3%)	3	-	-	3	3	-	-	-	-
11	*Actinomyces europaeus* (2%)	2	-	1	1	1	-	-	1	-
12	*Corynebacterium aurimucosum* (2%)	2	-	-	2	2	-	-	-	-
13	*Staphylococcus capitis* (2%)	2	1	1	-	2	-	-	-	-
14	*Corynebacterium simulans* (2%)	2	-	2	-	2	-	-	-	-
15	*Brevibacterium celere* (1%)	1	-	1	-	1	-	-	-	-
16	*Corynebacterium coyleae* (1%)	1	-	-	1	1	-	-	-	-
17	*Escherichia coli* (1%)	1	-	-	1	-	-	1	-	-
18	*Klebsiella pneumoniae* (1%)	1	-	-	1	-	-	-	1	-
19	*Micrococcus luteus* (1%)	1	-	1	-	1	-	-	-	-
*20*	*Acinetobacter nosocomialis* (1%)	1	-	-	1	1	-	-	-	-

**Table 2:** Distribution of virulence factors in the most prevalent bacterial species isolated.

No	Soluble Virulence Factors	Bacterial Species									
		*Staphylococcus* sp. Strains (*n* = 56)		*Corynebacterium* sp. Strains (*n* = 16)		*Enterococcus* Strains (*n* = 10)		*Proteus* sp. Strains (*n* = 3)		*Actinomyces* sp. Strains (*n* = 2)	
		24 h	72 h	24 h	72 h	24 h	72 h	24 h	72 h	24 h	72 h
1	α-hemolysin	22	27	3	5	0	1	0	0	0	0
2	β-hemolysin	7	8	0	0	0	0	0	0	0	0
3	γ-hemolysin	24	18	8	7	9	8	1	1	0	0
4	Caseinase	4	6	5	6	6	6	0	0	0	0
5	Amylase	0	7	0	0	0	1	0	1	0	0
6	Esculinase	8	9	2	2	8	9	0	0	0	0
7	Lipase	17	23	3	3	3	5	1	1	1	0
8	Lecithinase	34	35	5	5	0	0	0	0	0	0
9	DN-ase	0	0	0	0	0	0	0	0	0	0
10	Gelatinase	0	0	0	0	0	0	0	0	0	0

**Table 3:** Results of the genotypic assessment of 17 virulence genes in *Staphylococcus aureus* strains from HS through Polymerase Chain Reaction uniplex/simplex tests. Abbreviations: *fnbA* gene coding, fibronectin adhesin A; *coag* gene, coagulase enzyme) and multiplex tests (*clfA* and *clfB* genes, bacterial surface adhesins; *fnbB* gene, fibronectin-binding protein B; *fib* gene, fibrinogen-binding protein; *bbp* gene, bone sialoprotein-binding protein; *ebpS*, elastin-binding protein; *luk-PV*, Panton–Valentine leukocidin; *hlg*, hemolysin genes; *tst*, toxic shock syndrome toxin gene; *sea*, *seb*, *sec*, *sed*, *see*, classical staphylococcal enterotoxin genes.

Virulence Genes of *Staphylococcus aureus* Strains																	
Strain Index	*fnbB*	*fib*	*clfA*	*clfB*	*fnbA*	*cna*	*coag*	*bbp*	*ebpS*	*luk-PV*	*hlg*	*tst*	*sea*	*seb*	*sec*	*sed*	*see*
1	0	0	0	0	1	0	0	0	0	0	1	0	0	0	0	0	0
22	0	0	1	0	1	0	0	0	1	0	1	0	0	0	0	0	0
32	0	1	1	0	1	0	0	0	1	0	1	0	0	0	0	0	0
34	1	0	1	0	1	0	0	0	0	0	1	0	0	0	0	0	0
36	0	1	1	0	1	0	0	0	1	0	1	0	0	0	0	0	0
39	0	1	1	0	1	0	0	0	0	0	0	0	0	0	0	0	0
44	0	1	1	0	1	0	0	0	0	0	0	0	0	0	0	0	0
45	0	0	1	0	1	0	0	0	0	0	1	0	0	0	0	0	0
47	0	0	1	0	0	0	0	0	0	0	0	0	0	0	0	0	0
56	0	1	1	0	0	0	0	0	0	0	0	0	0	0	0	0	0
59	0	0	1	0	0	0	0	0	0	0	0	0	0	0	0	0	0
62	0	1	1	0	1	0	0	0	1	0	1	0	0	0	0	0	0
64	0	0	1	0	1	0	0	0	0	0	1	1	0	0	0	0	0
68	0	1	1	0	1	0	0	0	0	0	1	0	0	0	1	1	0
72	1	1	1	0	1	0	0	0	1	0	1	0	0	0	0	0	0
77	1	1	1	0	1	0	0	0	0	0	1	0	0	0	0	0	0
86	0	1	1	0	1	0	0	0	1	0	1	0	0	0	0	0	0
88	0	1	1	0	1	0	0	0	0	0	0	0	0	0	0	0	0
92	1	1	1	0	1	0	0	0	1	0	1	0	0	0	0	0	0
94	0	1	1	0	1	0	0	0	1	0	1	0	0	0	0	0	0
100	0	1	1	0	1	0	0	0	1	0	1	0	0	0	0	0	0
107	0	0	1	0	0	0	0	1	0	0	0	0	0	0	0	0	0
108	0	1	1	0	1	0	0	1	0	0	0	0	0	0	0	0	0
125	0	1	1	0	1	0	0	0	0	0	1	0	0	0	0	0	0
130	0	0	1	0	1	0	0	0	0	0	1	0	0	0	0	0	0
138	0	1	1	0	1	0	0	0	1	0	1	0	0	0	0	0	0
179	0	0	1	0	1	0	0	0	0	0	1	0	0	0	0	0	0
203	0	1	1	0	1	0	0	0	1	0	1	0	0	0	0	0	0
204	0	1	1	0	1	0	0	0	1	0	1	0	0	0	0	0	0
205	0	1	1	0	1	0	0	0	1	0	1	0	0	0	0	0	0
218	1	1	1	0	1	0	0	0	0	0	1	0	0	0	0	0	0
229	0	1	1	0	1	0	0	0	1	0	1	0	0	0	0	0	0
253	0	1	1	0	1	0	0	0	1	0	1	0	0	0	1	0	0
254	0	1	1	0	1	0	0	0	1	0	1	0	0	0	1	0	0
255	0	1	1	0	1	0	0	0	0	0	0	0	0	0	0	0	0

**Table 4:** Results of genotypic assessment of 20 resistance genes in *Staphylococcus aureus* strains from HS through PCR tests. Abbreviations: *mecA* gene, encoding penicillin-binding protein 2a; *nuc*, encoding thermonuclease; *ermA* and *ermC*, encoding resistance genes for erythromycin; Staphylococcal cassette chromosome group, SCC*mec* group, *ccrC* and *ccrB2*, cassette chromosome recombinase; *Kdp*, high-affinity K^+^ transporter.

Strain Index	mecA	nuc	aac-aph	ermA	ermC	CIF2	ccrC	ccrB2	sccmec IV	sccmec III	kdp	deS	Type IV a	Type II	Type IV b	Type IV c	Type IV d	Type I	Type V	Type III
32	1	1		0	1	1	0	1	0	0	0	0	0	0	0	0	0	0	0	0
34	0	0	1	0	0	0	0	0	0	0	0	0	0	0	0	0	0	0	0	0
36	0	0	1	0	0	0	0	0	0	0	0	0	0	0	0	0	0	0	0	0
39	1	1	0	0	0	1	0	1	0	0	0	0	0	0	0	0	0	0	0	0
44	1	1	0	0	0	1	0	1	0	0	0	0	0	0	0	0	0	0	0	0
59	0	0	0	0	0	0	0	0	0	0	0	0	0	0	0	0	0	0	0	0
62	0	0	0	0	1	0	0	0	0	0	0	0	0	0	0	0	0	0	0	0
68	1	1	0	0	0	1	0	1	0	0	0	0	0	0	0	0	0	0	0	0
86	1	1	0	0	0	1	0	1	0	0	0	0	0	0	0	0	0	0	0	0
88	1	1	0	0	1	0	0	0	0	0	0	0	0	0	0	0	0	0	0	0
94	0	0	1	0	0	0	0	0	0	0	0	0	0	0	0	0	0	0	0	0
100	0	0	0	0	0	0	0	0	0	0	0	0	0	0	0	0	0	0	0	0

**Table 5:** Study inclusion criteria.

No.	Inclusion Criteria
1	Female or male patients aged from 18 to 70 years.
2	The subject must have HS skin lesions for at least 1 year.
3	Show at least one anatomical region with inflammatory (active) lesions.
4	Patients from Hurley stage I, II, and III were included in this study.

**Table 6:** Study exclusion criteria.

No.	Exclusion Criteria
1	The patient has any other active disease (e.g., infections) that may interfere with the result of bacteriological investigations.
2	Subject administered topical treatments (except local antiseptics) 30 days prior to collection of bacteriological samples.
3	The subject received systemic antibiotic treatment during the past month before the collection of bacteriological samples.

## Data Availability

The datasets generated and analyzed during the current study are available from the corresponding author upon reasonable request.
